# Estimating birthweight reduction attributable to maternal ozone exposure in low- and middle-income countries

**DOI:** 10.1126/sciadv.adh4363

**Published:** 2023-12-08

**Authors:** Mingkun Tong, Huiyu Xu, Ruohan Wang, Hengyi Liu, Jiajianghui Li, Pengfei Li, Xinghua Qiu, Jicheng Gong, Jing Shang, Tong Zhu, Tao Xue

**Affiliations:** ^1^ Institute of Reproductive and Child Health, National Health Commission Key Laboratory of Reproductive Health and Department of Epidemiology and Biostatistics, Ministry of Education Key Laboratory of Epidemiology of Major Diseases (PKU), School of Public Health, Peking University Health Science Center, Beijing, China.; ^2^ Center for Reproductive Medicine, Department of Obstetrics and Gynecology, Peking University Third Hospital, Beijing 100191, China.; ^3^ Advanced Institute of Information Technology, Peking University, Hangzhou, Zhejiang, China.; ^4^ Institute of Medical Technology, Peking University Health Science Center, Beijing, China.; ^5^ SKL-ESPC and SEPKL-AERM, College of Environmental Sciences and Engineering, and Center for Environment and Health, Peking University, Beijing 100871, P. R. China.; ^6^ State Environmental Protection Key Laboratory of Atmospheric Exposure and Health Risk Management and Center for Environment and Health, Peking University, Beijing, China.

## Abstract

The effect of O_3_ on birthweight in low- and middle-income countries (LMICs) remains unknown. A multicenter epidemiological study was conducted to evaluate the association between maternal peak-season O_3_ exposure and birthweight, using 697,148 singleton newborns obtained in 54 LMICs between 2003 and 2019. We estimated the birthweight reduction attributable to peak-season O_3_ exposure in 123 LMICs based on a nonlinear exposure-response function (ERF). With every 10–part per billion increment in O_3_ concentration, we found a reduction in birthweight of 19.9 g [95% confidence interval (CI): 14.8 to 24.9 g]. The nonlinear ERF had a monotonic decreasing curve, and no safe O_3_ exposure threshold was identified. The mean reduction in birthweight reduction attributable to O_3_ across the 123 LMICs was 43.8 g (95% CI: 30.5 to 54.3 g) in 2019. The reduction in O_3_-related birthweight was greatest in countries in South Asia, the Middle East, and North Africa. Effective O_3_ pollution control policies have the potential to substantially improve infant health.

## Teaser

In LMICs, maternal O_3_ exposure can notably reduce birthweight, particularly in South Asia, the Middle East and North Africa.

## INTRODUCTION

Birthweight is an important indicator of fetal growth. Low birthweight (LBW), defined as a weight at birth of <2500 g, is associated with maternal malnutrition, maternal diseases, and poor health care during pregnancy. LBW increases the likelihood of neonatal, infant, all-cause, and cardiovascular mortalities during adulthood (*1*, *2*). LBW is the most commonly used metric of child health and nutritional status (*3*). In 2015, the worldwide prevalence of LBW was estimated as 14.6% (range: 12.4 to 17.1%). In total, there were approximately 20.5 million (range: 17.4 million to 24.0 million) LBW newborns, 91% of whom were born in low- and middle-income countries (LMICs), mainly South Asia (48%) and sub-Saharan Africa (24%) (*4*). Although several behavioral and biological risk factors for LBW, including multiple births, pregnancy-induced hypertension, low pre-pregnancy body mass index (BMI), smoking, cocaine use during pregnancy, and history of preterm birth (PTB) (*5*), have been established, the etiology of LBW is not fully understood.

Ambient air pollution appears to play an important role in fetal development. For example, in a global study, every 10 μg/m^3^ increment in ambient fine particulate matter (PM_2.5_) was associated with a reduction in birthweight of 22 g [95% confidence interval (CI): 12 to 32 g], and a 30-g reduction in birthweight was directly attributed to outdoor PM_2.5_ exposure (*6*). However, no study has assessed the global or regional impact of ambient ozone (O_3_) on birthweight; the need for such assessments is particularly urgent in LMICs, such as Middle East Countries, where LBW rates and O_3_ pollution levels tend to be high (*7*). The lack of representative exposure-response functions (ERFs) for LMICs is the main barrier to determine the effect of O_3_ on birthweight. In addition, more than half of newborns in LMICs are not weighed at birth, particularly in South Asia and sub-Saharan Africa, which makes it difficult for epidemiological studies to derive ERFs in those regions (*8*). Thus, current evidence of an association between O_3_ and birthweight is largely limited to high-income countries. A meta-analysis reported that O_3_ exposure throughout the entire pregnancy was associated with a birthweight reduction of 4.6 to 27.3 g for every 10–part per billion (ppb) increment in O_3_ (*9*). Similarly, studies performed in the United States, China, and Korea reported birthweight reductions of 5.7 to 7.9 g per 10-ppb increment in O_3_ exposure (*10*–*12*).

To fill the gap, a large epidemiological study is needed to confirm the effect of O_3_ exposure on birthweight in LMICs. Therefore, we performed a multicenter epidemiological study of 697,148 singleton newborns in 54 LMICs and evaluated the association between maternal O_3_ exposure and birthweight. Furthermore, we developed a representative ERF and calculated the O_3_-related birthweight in 123 LMICs that covered approximately 90% of all LBW newborns worldwide.

## RESULTS

### Descriptive statistics

The mean ± SD maternal age was 26.0 ± 6.0 years. In total, 359,863 (51.6%) of the 697,148 singleton live births were boys. The mean birthweight was 3061 ± 678.6 g. The spatial distribution of the study population was shown in fig. S1. A total of 272,741 (39.1%) women were from India, and the rest were from other 53 countries (table S1). Most of the women resided in sub-Saharan Africa [*n* = 284,902 (40.9%)] and South Asia [*n* = 282,147 (40.5%)]; the rest lived in Latin America and the Caribbean [*n* = 48,063 (6.9%)], the Middle East and North Africa [*n* = 39,031 (5.6%)], East Asia and the Pacific [*n* = 22,154 (3.2%)], and Europe and Central Asia [*n* = 20,851 (3.0%)]. The mean peak-season O_3_ exposure amount was 52.67 ± 10.35 ppb, with a considerable variation between different regions. Figure S1 shows that O_3_ pollution was most and least severe in North India and the Caribbean, respectively. Tables S2 and S3 provide more detailed description of population characteristics and exposure levels, respectively.

### Association between O_3_ and birthweight

**Fig. 1.**
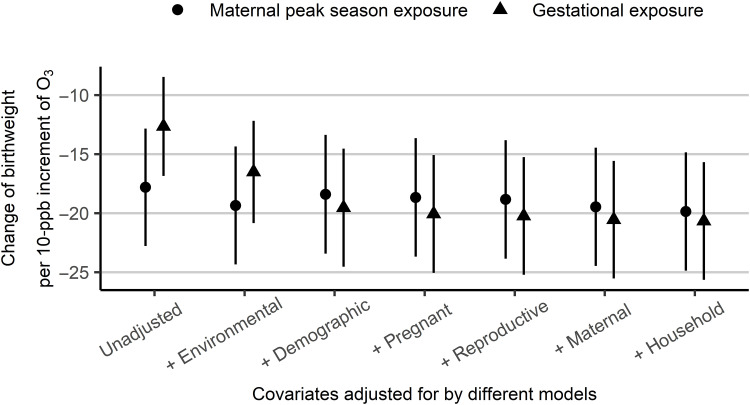
Linear association between ozone (O_3_) exposure and birthweight estimated by different models. The adjusted covariates are (i) environmental variables (PM_2.5_ and temperature), (ii) birth characteristics (sex and month of birth × latitude zones), (iii) pregnancy variables (cesarean section, place of delivery, antenatal care attendance, and nulliparous or not), (iv) variables related to reproductive history (maternal age and inter-pregnancy interval), (v) maternal variables (maternal BMI and maternal employment status), and (vi) household features (sex and age of household head, source of drinking water, type of toilet, and type of cooking energy).

The effect of O_3_ on birthweight did not differ after adjusting for covariates and did not vary considerably according to the exposure assessment method (Fig. 1), outcome measure (fig. S2), or subpopulation (fig. S3). In the unadjusted model, every 10-ppb increment in maternal peak-season O_3_ exposure was associated with a reduction in birthweight of 17.8 g (95% CI: 12.8 to 22.8 g). In the fully adjusted model, the birthweight reduction was estimated as 19.9 g (95% CI: 14.8 to 24.9 g), which was comparable with that estimated based on gestational O_3_ exposure (20.7 g; 95% CI: 15.7 to 26.7 g). Similarly, O_3_ exposure had a significant negative effect on birthweight in the analysis of the alternative O_3_ dataset with a fine spatial resolution (0.1° × 0.1°) and low temporal resolution. However, the point estimate method yielded a smaller reduction of 6.1 g (95% CI: 2.4 to 9.8 g; fig. S2A). In addition, in the analysis including birthweight as a dichotomous variable (<2500 g versus ≥2500 g), every 10-ppb increment in maternal peak-season O_3_ exposure was associated with a 4% (95% CI: 1 to 6%) increase in the odds of LBW (fig. S2B). Last, O_3_ exposure and birthweight consistently showed an association in subgroup analyses; the relationship was strongest in sub-Saharan Africa and South Asia (fig. S3).

### Nonlinear ERF

**Fig. 2.**
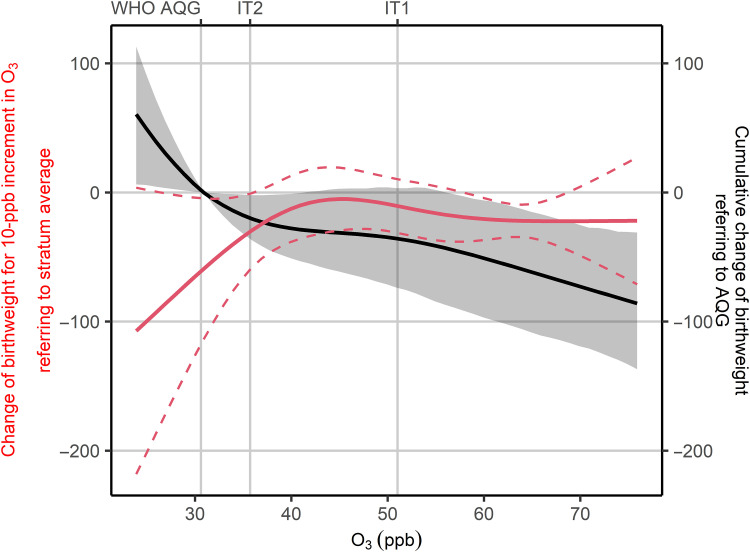
Nonlinear ERF of maternal peak-season O_3_ exposure and birthweight. The stratum-specific marginal effect (solid red line) and corresponding 95% CIs (dashed red line) are evaluated as the change of birthweight per 10-ppb increment of O_3_, given different stratum-level average O_3_ concentrations (left *y* axis). Strata were sampling units determined before conducting DHS fieldworks. The estimated effects are based on linear associations between within-stratum variations in exposure and those in birthweight, and thus reflect first derivatives of the ERF. An integration of the marginal effects can obtain the ERF (black line) and corresponding 95% CI (gray ribbon). We set the TMREL equal to the AQG (60 μg/m^3^) recommended by WHO.

Using a fully adjusted model, we derived nonlinear associations between peak-season O_3_ exposure and birthweight (Fig. 2). Generally, the nonlinear ERF showed a sublinear decreasing curvature when air quality guideline (AQG) was used as the reference (black line). According to the first derivatives of the function, O_3_-related birthweight reduction was observed along the full range of O_3_ concentration (solid red line). The adverse effect attenuated gradually at the high concentration. Surrounding AQG and interim target 2 (IT2), a marginal 10-ppb increment was associated to a reduced birthweight of 59.2 g (95% CI: 4.6 to 114 g) and 29.4 g (95% CI: 0.58 to 58.2 g), respectively. On the basis of the pointwise CIs, between IT1 and IT2, the marginal effect was not significant, suggesting that the overall effect increased to a plateau stage. Above IT1, the negative effect of O_3_ exposure on birthweight was stable; a 10-ppb increment in O_3_ was associated with a reduction in birthweight of approximately 22 g. It suggests that the substantial health benefit (i.e., birthweight increase) would be achieved when reducing O_3_ pollution levels from above IT1 to IT1 and from below IT2 to a lower level.

Given the sublinear decreasing ERF, we were unable to identify a safe level of O_3_ exposure with respect to birthweight. Because most of the study population was exposed to O_3_ levels exceeding the AQG, we used it as the theoretical minimum risk exposure level (TMREL). Comparing to an alternative ERF using IT2 as TMREL, the main model had higher degrees of uncertainty at the high-concentration levels. However, using different TMRELs did not change the curvature of the estimated ERF.

### Attributable burden of birthweight reduction

**Fig. 3.**
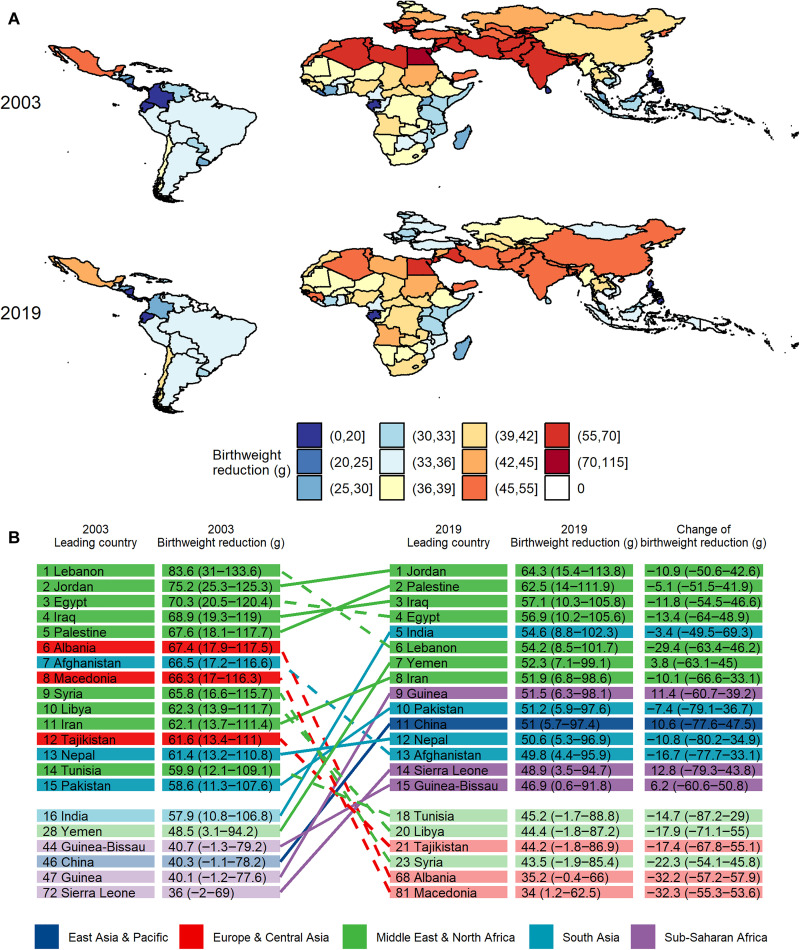
The birthweight reduction attributable to maternal peak-season ozone exposure in 2003 and 2019. Map of 123 low- and middle-income countries (**A**) and the leading 15 countries (**B**). The estimates are based on the ERF for the minimum risk exposure level of WHO AQG (i.e., 60 μg/m^3^).

We estimated the birthweight reduction attributable to peak-season O_3_ exposure in 123 LMICs. Between 2003 and 2019, the number of LBW newborns gradually increased from 11.1 million to 12.5 million, whereas **E**_*i*,*y*_ barely changed (52.5 and 52.3 ppb in 2003 and 2019, respectively; fig. S4A). During this period, >99.7% of LBW newborns were in regions with O_3_ concentrations exceeding the AQG (fig. S4B). In 2003 and 2019, the mean birthweight reductions attributable to peak-season O_3_ exposure were 44.7 g (95% CI: 31.1 to 56.7 g) and 43.8 g (95% CI: 30.5 to 54.3 g), respectively; the corresponding relative reductions were 1.40% (95% CI: 0.97 to 1.77%) and 1.39% (95% CI: 0.96 to 1.72%; fig. S4C). Figure 3A shows the spatial distribution of O_3_-related birthweight reductions among the 123 countries. Throughout the study period, countries in South Asia, the Middle East, and North Africa, such as Egypt, Iraq, Iran, India, Pakistan, and Afghanistan, were always the hotspots of the attributable burden of birthweight reduction due to the highest O_3_ concentrations. For three countries in Europe and Central Asia (Albania, Macedonia, and Tajikistan), the attributable burden of birthweight reduction was substantially reduced between 2003 and 2019, whereas for three countries in the Atlantic Ocean and West Africa group (Guinea, Sierra Leone, and Guinea-Bissau) and one in East Asia (China), the burden increased substantially (Fig. 3B).

Compared to the absolute birthweight reduction, relative reduction might be a more indicative index to predict subsequent health outcomes. For instance, although the absolute birthweight reduction was comparable among regions, the relative reduction was higher in South Asia than in the Middle East and North Africa, which could be attributed to the lower baseline birthweight of Asian newborns (fig. S5). Given that, the O_3_-related birthweight reduction might have a pronounced health impact in South Asia. In addition, the attributable burden in the East Asia and Pacific region increased between 2016 and 2019; the relative reductions in birthweight in these years were 1.1% (35.4 g) and 1.3% (42.4 g), respectively. The increasing level of O_3_ pollution in China between 2016 and 2019 may partially explain these results (*13*, *14*). Although geographic differences in the burden of O_3_-related birthweight reduction have lessened during the past two decades, pollution levels are still increasing in some countries.

**Fig. 4.**
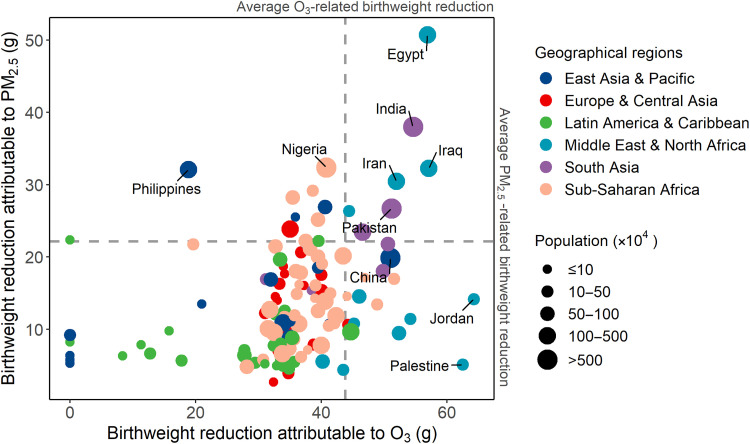
Comparison of the birthweight reduction attributable to ozone (O_3_) exposure in the current study to the well-developed estimates on birthweight reduction attributable to PM_2.5_ in 123 low- and middle-income countries in 2019.

Overall, we found that O_3_ exposure was a considerable risk factor of birthweight reduction, even compared to the PM_2.5_. We compared the Global Burden of Disease (GBD) Collaborative Network estimates of PM_2.5_-related birthweight reductions with our O_3_-related birthweight reduction data for 2019 (Fig. 4). Across all 123 LMICs, the mean reduction in birthweight attributable to O_3_ was estimated as 43.8 g (95% CI: 30.5 to 54.3 g) and 22.1 g (95% CI: 21.9 to 22.3 g) due to maternal exposure to O_3_ and PM_2.5_, respectively. We classified all countries into four groups according to the impact of air pollutants on birthweight (high versus low PM_2.5_- and O_3_-related burdens). For countries such as China, Jordan, and Palestine, birthweight reductions were attributable mainly to O_3_ exposure, whereas PM_2.5_ exposure was more important in countries, such as the Philippines. For populous LMICs in the Middle East and South Asia (i.e., Egypt, Iraq, Iran, India, and Pakistan), both O_3_ and PM_2.5_ were important.

## DISCUSSION

We analyzed Demographic and Health Surveys (DHS) Program data for 54 LMICs and observed a robust negative association between O_3_ exposure and birthweight: Birthweight was 19.9 g lower for every 10-ppb increment in maternal peak-season O_3_ exposure. Moreover, the ERF showed a sublinear decreasing trend, and no safe O_3_ exposure threshold was identified. We also estimated the burden of O_3_-related birthweight reduction in 123 LMICs. In 2019, the mean reduction in birthweight attributable to O_3_ exposure was estimated as 43.8 g (95% CI: 30.5 to 54.3 g), which corresponded to 1.39% (95% CI: 0.96 to 1.72%) of the baseline birthweight. The burden was heaviest in South Asia, Middle East, and North Africa.

The negative association between O_3_ exposure and birthweight found in this study accords with the literature. Sun *et al.* (*10*) analyzed more than 2 million singleton term births in the United States and found that each 10-ppb increment in O_3_ during the pregnancy was associated with a reduction in birthweight of 7.6 g (95% CI: 6.4 to 8.8 g) and increasing odds of small for gestational age (SGA) by 3% (95% CI: 2 to 4%). Similarly, Guo *et al.* (*11*) conducted a study in China including more than 2.5 million singleton term births. They reported a 5.67-g (95% CI: 1.31 to 10.0 g) reduction in birthweight and 4.8% (95% CI: 0 to 8.2%) increase in odds of SGA. Lamichhane *et al.* (*12*) examined the effect of O_3_ exposure during the third trimester and reported a birthweight reduction of 7.88 g (95% CI: 0.35 to 15.4 g). Compared with previous studies, our study obtained an effect of larger size from the model of linear association between O_3_ and birthweight. This inconsistency could be partially explained by differences in exposure level and covariate adjustment. First, the average of gestational O_3_ concentrations in our study was 46.4 ppb, which was higher than that by Sun *et al.* (*10*) (i.e., 39 to 40 ppb) or Lamichhane *et al.* (*12*) (43.0 ppb). The linear association can be viewed as an average of marginal stratum-specific effects. According to our nonlinear analysis (Fig. 2), the linear estimates could be modified by distributions of sample exposure levels, which were different between studies. Second, birthweight is determined mainly by the duration of gestation and rate of fetal growth. Therefore, previous studies have explained the risk of LBW by PTB and intrauterine growth retardation, indicated by the SGA. It was important to note that some previous studies on health effects of air pollution included term singleton live births and adjusted for the gestational age in their regression models. This approach allowed them to focus solely on the direct effect of O_3_ on birthweight rather than the mediated effect by short gestation. Because of the lack of gestational age, our models happened to investigate the overall effect and thus resulted in an estimated effect of larger size, compared to those from previous studies.

Notably, our study did not account for gestational age in analysis due to the lack of such information in DHS database, making our results limited in following three aspects. First, the absence of gestational age could lead to exposure misclassification in temporal dimension, because of applying a uniform time window to assess gestational exposure. To partially address this, we defined four distinct exposure metrics in sensitivity analysis, and the consistency in the estimated associations suggested that our findings were robust given the potential misclassification. Second, we could not derive some adverse birth outcomes such as PTB and SGA, without data on gestational age. As a result, we could not further investigate how the O_3_-birthweight association was mediated by short gestation or intrauterine growth retardation, which had been identified as two major pathways underlying the etiology of birthweight reduction. Particularly, a large proportion of newborns in LMICs were affected by SGA but not short gestation (*15*, *16*). Determining whether SGA or PTB predominately contributes to birthweight reduction across different regions is essential for public health interventions. Furthermore, the two pathways might play distinguishable roles at different trimesters, which resulted in one or several susceptible time windows to O_3_ exposure identified by previous studies (*10*, *12*, *17*). Future research should explore these biological pathways underlying the burden of O_3_-related birthweight reduction using data with advanced quality. Third, live birth bias was also a concern, also related to the gestational age. Specifically, because O_3_ exposure and short gestational age can independently increase the likelihood of pregnancy loss, fraction of PTBs among the high-exposure live births is lower than that among the low-exposure live births, due to different survival rates. Given that, ignoring live birth bias leads to a confounded association between low O_3_ exposure and LBW. Therefore, the effect might be underestimated by our study, which did not incorporate gestational age to address the live birth bias.

LBW is a major public health issue worldwide. Although the global prevalence of LBW decreased from 17.5% in 2001 to 14.6% in 2015, the average annual rate of reduction of 1.23% is below the rate that will be required (i.e., 2.74%) to achieve a 30% reduction in the proportion of LBW newborns between 2012 and 2025 [Global Nutrition Target 3 of the World Health Organization (WHO)] (*3*, *4*). In South Asia, there were almost 10 million LBW newborns in 2015, and the average annual rate of reduction was only 1.37% (*4*). In addition to infant and child mortality, LBW is associated with numerous short- and long-term diseases (*2*, *18*–*22*). Interventions to address LBW may aid the achievement of Sustainable Development Goal 3, which is to “Ensure healthy lives and promote well-being for all at all ages” (*23*). Our findings suggest that controlling O_3_ pollution may increase the likelihood of achieving Global Nutrition Target 3 and Sustainable Development Goal 3.

According to the State of Global Air 2020 report, worldwide O_3_ exposure increased from 47.3 ppb in 2010 to 49.5 ppb in 2019 (*7*). The concentration of O_3_ is steadily increasing because of higher emissions of its chemical constituents and ongoing global warming. Therefore, the negative health effects of O_3_ will also continue to increase in the absence of effective policy interventions. Long-term exposure to O_3_ is a risk factor for adult mortality and cardiorespiratory diseases (*24*, *25*). Moreover, prenatal exposure to O_3_ is associated with a higher risk of stillbirth and PTBs (*26*–*28*). Unlike PM_2.5_, O_3_ pollution is invisible such that the general public is often unaware of its presence and adverse health effects, particularly on reproductive and child health. Our results suggest that the burden of O_3_-related birthweight reduction is considerable; O_3_ even had a greater adverse effect on birthweight than PM_2.5_ (*6*). As an underestimated and invisible risk factor, controlling for O_3_ pollution should be paid additional attentions to protect reproductive health and child health.

Besides the absences of gestational age, this study had several other limitations. First, the spatial exposure misclassification was unavoidable. Because of a lack of residential address data, individual O_3_ exposure levels were based only on the general geographical location. We were also unable to estimate exposure according to daily activities and could not consider the impact of migration during pregnancy. Moreover, the global monthly O_3_ concentration data had a coarse spatial resolution, which could also contribute to exposure misclassification. Using the 0.5° × 0.5° concentrations instead of the 0.1° × 0.1° concentrations did not clearly reduced the fraction of within-stratum variations in exposure (table S4), which was associated to the within-stratum variations in birthweight to derive the ERF. Although correctly specifying peak-season O_3_ by monthly concentrations at the sacrifice of spatial resolution did not considerably reduce statistical power of our multicenter study, the relevant biasness should be quantified by future studies with advanced methods to evaluate exposure misclassification. Second, our outcome, birthweight, was not clinically measured but self-reported by mothers, which can lead to reporting bias. In LMICs, where a sizeable proportion of newborns are delivered at home, birthweight is not always measured (*29*). However, we treated birthweight as a continuous rather than dichotomous variable to reduce the likelihood of outcome misclassification. Third, we did not adjust for the precursors of O_3_ (e.g., nitrogen oxides and volatile organic compounds) in the model because of the lack of suitable data covering the current study regions and study period. Because the estimated burden might be attributable to its precursors but not to O_3_ itself, our findings should be interpreted carefully. Fourth, because the fixed effects only controlled unmeasured confounders in strata level, existence of residual confounders varying within a stratum could not be completely avoided by covariates adjustment. Last, we considered only uncertainties in the ERF in our risk assessment. Ideally, uncertainties associated with the estimates of the O_3_ exposure concentration would also have been taken into account.

In conclusion, according to our analysis of 697,148 singleton newborns in 54 LMICs, maternal exposure to O_3_ can have a major impact on birthweight. Among 123 LMICs, the mean birthweight in 2019 was reduced by 43.8 g (95% CI: 30.5 to 54.3 g) due to O_3_ exposure. The burden of O_3_-related birthweight reduction was particularly high in South Asia, Middle East, and North Africa. Reducing O_3_ pollution could improve reproductive and child health in LMICs.

## METHODS

### Study population

We extracted data from the DHS Program (https://dhsprogram.com/), which used a two-step procedure to collect nationally representative data. In the first stage, primary sampling units, usually census enumeration areas, were selected with probability proportional to size according to geographic region and area type (i.e., urban versus rural). In the second stage, 25 to 30 households within each primary sampling unit were sampled; all households had the same probability of being sampled. Mothers aged 15 to 49 years were asked about the health, nutrition, and survival status of their children born within the 5-year period preceding the survey. The DHS program assigned weights to households and individual women according to their sampling probabilities and response rates; this increased the representativeness of the data at the population level within each stratum. The geographic locations of the respondents were recorded using global positioning systems. Procedures and questionnaires for DHS surveys have been reviewed and approved by ICF Institutional Review Board. This study was based on the publicly available DHS data and adhered to its data usage guidelines. No further ethic approval was required.

Because of the limited availability of O_3_ concentration data, we could analyze geocoded survey data only during the period 2003–2019; data obtained during 114 surveys that recorded birth history were included in our analysis. We excluded records indicating potential stillbirth (i.e., records of infants with the same birth and death dates), as well as those lacking birthweight data and those of non-singleton births. The final dataset included 697,148 singleton newborns in 54 LMICs (*n* = 2272 sampling strata).

### Exposure assessment

We obtained O_3_ data from a global dataset of monthly surface O_3_ concentrations with a more complete spatiotemporal coverage (*30*); this O_3_ dataset was constructed by applying ensemble machine learning techniques to satellite observations, chemical transport model outputs, atmospheric reanalysis and emission data, and ground-surface observations. The monthly mean daily maximum 8-hour average (MDA8) O_3_ concentrations during 2003–2019 were calculated across a regular 0.5° × 0.5° (≈50 km × 50 km) grid. The overall accuracy was high (*R*^2^ = 0.917), and the deviance was low (root mean square error = 4.16 ppb). We distinguished two types of O_3_ exposure. First, maternal peak-season O_3_ exposure was calculated as the maximum 6-month running average of monthly means of MDA8 in the six consecutive months for the entire year (i.e., the prior 12 months) before birth, according to the long-term O_3_ exposure limit of the AQGs released by the WHO in 2021. Second, we calculated gestational O_3_ exposure (i.e., exposure during pregnancy). In >90% of all DHS, gestational age data were not recorded; >90% of the women for whom these data were reported had a gestational age of 9 months. Therefore, we calculated the monthly mean MDA8 for the 9-month period preceding birth.

An alternative O_3_ concentration product with a finer spatial resolution (0.1° × 0.1° ≈ 10 km × 10 km) and coarser temporal resolution (yearly) was obtained from the GBD Collaborative Network (*31*). We also calculated the maternal peak-season and gestational O_3_ exposure based on this alternative O_3_ product. The maternal peak-season O_3_ exposure was presented as the peak-season concentration of O_3_ at the year of birth, and thus was named as “yearly O_3_ exposure.” Gestational exposure corresponded to the weighted mean of the concentration in two consecutive years (i.e., the calendar year of birth plus the one preceding it) and was referred to herein as the “gestation-weighted O_3_ exposure.” For example, if a live birth was born in May 2010, the gestation-weight O_3_ exposure level was calculated as ^5^/_9_ × O_3_ concentration in 2010 + ^4^/_9_ × O_3_ concentration in 2009. Last, to control for environmental confounders, we obtained monthly temperatures (*32*) and PM_2.5_ concentrations (*33*) from global products. The two variables were prepared in the same way as O_3_.

### Covariates

O_3_ concentrations, co-determined by methodological conditions and anthropogenic emissions such as those from traffic, are correlated with climate variables and individual characteristics that can be directly or indirectly affected by the socioeconomic status. Therefore, the covariates included sex of the newborn (boy or girl), cesarean section (yes or no), place of delivery (home, hospital, private, or others), antenatal care attendance (yes or no), parity (nulliparous or multiparous), maternal age (≤23 years, 24 to 28 years, or ≥29 years), inter-pregnancy interval (none, 1 to 35 months, or ≥36 months), maternal BMI (underweight, normal, overweight, or obese), maternal employment (yes or no), sex of household head (male or female), age of household head (≤35 years, 36 to 50 years, or ≥51 years), source of drinking water (bottled, natural, piped, rain, tank, tube, well, or others), type of toilet (composting, flush, pit, none, or others), and main energy source for cooking (agricultural crop, animal dung, biogas, charcoal, lignite coal, electricity, kerosene, liquefied petroleum gas, natural gas, straw, shrubs or grass, wood, or others). The missing values of covariates were randomly imputed by chained equations using the R package mice (*34*).

### Statistical analyses

On the basis of the sampling design of the DHS program, we used a fixed-effects linear regression model to analyze the association between maternal peak-season O_3_ exposure and birthweight. The sampling strata used in the DHS program [i.e., stratification according to region and area type (urban versus rural)] were treated as nuisance parameters and included as fixed effects. Each individual was assigned a weight to enhance the representativeness of the data within each stratum, as described above; these weights were then incorporated into the regression models to adjust for sampling bias. We also adjusted for PM_2.5_, temperature, and the covariates mentioned above. Seasonality was also considered as a potential confounder, and a categorical variable was created to account for the effect of the interaction between month of birth (i.e., 1 to 12) and zone of latitude (<0°, 0° to 23.5°, or >23.5°). To account for the potential nonlinear effects of temperature, maternal age, pregnancy interval, and age of the head of the household, all of these variables were modeled as spline functions with three degrees of freedom.

The main outcome variable was the change in birthweight per 10-ppb increment in O_3_ exposure; we performed multiple sensitivity analyses to test the robustness of this association. First, in addition to maternal peak-season O_3_ exposure, we analyzed the effects of gestational O_3_ exposure, yearly O_3_ exposure, and gestation-weighted O_3_ exposure on birthweight. Second, we replaced birthweight with a binary variable (birthweight <2500 or ≥2500 g) and reevaluated the associations using logit regression. Last, to examine the potential modifiers on the association, we conducted interaction analyses using the subpopulation indicators of geographical regions, income, sex of the newborn, cesarean section, place of delivery, antenatal care attendance, nulliparous status, maternal age, pregnancy interval, maternal BMI, maternal employment, sex and age of the head of the household, source of drinking water, type of toilet, and main energy source for cooking food. The statistical significance of the interaction between each modifier and O_3_ was determined by the Wald test.

Using a varying-coefficient regression model, we estimated how the marginal effect of within-stratum variation in O_3_ concentrations varies with different stratum-specific averages of O_3_ concentrations (first derivative of the ERF). Next, given a predetermined TMREL, we applied an integration to obtain the effect for an increase from TMREL to a given level of O_3_ concentration (nonlinear ERF). The statistical methods were described in more detail in our recently published work and were developed specifically for this multicenter epidemiological study (*35*).

### Risk assessment

To assess spatial and temporal variations in O_3_-related birthweight reductions during 2003–2019, we conducted a risk assessment across 123 LMICs (there are 140 LMICs in total; 17 island countries were excluded from the analysis, as described in the Supplementary Materials) based on the ERF. First, we calculated the weighted average peak-season O_3_ concentration (**E**_*i*,*y*_) for each country and year using the following equation

**E**_*i*,*y*_ = (∑_*s*∈*i*_ [**C**_*s*,*y*_ × ∑*_k_* **P**_*s*,*y*,*k*_])/(∑_*s*∈*i*_∑*_k_* **P**_*s*,*y*,*k*_)

where *s*, *k*, *i*, and *y* represent spatial pixels, sex, country, and year, respectively; **C**_*s*,*y*_ is the gridded annual peak-season O_3_ concentration (spatial resolution = 0.5° × 0.5°); and **P**_*s*,*y*,*k*_ is the gridded annual sex-specific population of 0 to 1 year olds, which was used to represent the number of newborns. Because the original spatial resolution of the WorldPop dataset was 1 km × 1 km, we transformed the data for compatibility with the 0.5° × 0.5° spatial resolution of our O_3_ dataset.

At the country-level, O_3_-related birthweight reductions were determined on the basis of **E**_*i*,*y*_ and the estimated ERF. Overall and region-specific O_3_-related birthweight reductions were calculated by averaging the country-level estimates, with weights applied according to the number of newborns. We also obtained country-level mean birthweight data from the GBD Collaborative Network for the period 2003–2017. Because estimates were not available for 2018 and 2019, we used the 2017 data for both of these years. Then, we calculated the relative birthweight reduction attributable to O_3_ exposure. Because of the high computational burden, we considered only uncertainties directly associated with the ERF. A Monte Carlo modeling approach was used to calculate 95% CIs.

All analyses were performed using R software (4.2.0; R Core Team, Vienna, Austria). Statistical inference was performed using the R packages fixest and mgcv.
