# Supplementary material for: Estimating birthweight reduction attributable to maternal ozone exposure in low- and middle-income countries
Source: Sci Adv. 2023 Dec 8;9(49):eadh4363. doi: 10.1126/sciadv.adh4363 (PMC10708175; doi:10.1126/sciadv.adh4363)
Supplement: Supplementary file 1 — Supplementary Text Tables S1 to S4 Figs. S1 to S5 [file sciadv.adh4363_sm.pdf]

Science Advances
AAAS

# Supplementary Materials for

## Estimating birthweight reduction attributable to maternal ozone exposure in low- and middle-income countries

Mingkun Tong *et al.*

Corresponding author: Tao Xue, txue@hsc.pku.edu.cn;Tong Zhu, tzhu@pku.edu.cn

*Sci. Adv.* **9**, eadh4363 (2023)
DOI: 10.1126/sciadv.adh4363

**This PDF file includes:**

Supplementary Text
Tables S1 to S4
Figs. S1 to S5

# Supplementary Text

**1. The selection of 123 low- and middle-income countries in the risk assessment.**

Among global 140 LMICs, due to the missing value of $O_3$ exposure data, 17 island countries were excluded, including American Samoa, Seychelles, Saint Vincent and the Grenadines, Saint Lucia, Mauritius, Maldives, Grenada, Dominica, Antigua and Barbuda, Federated States of Micronesia, Marshall Islands, Tonga, Samoa, Kiribati, São Tomé and Príncipe, Cape Verde, Comoros.

**Table S1.** Surveys and their sample sizes included in the current study.

| ISO | DHS country code | Survey | Sample size |
|---|---|---|---|
| ALB | AL | Albania Standard DHS, 2008-09 | 1427 |
| | | Albania Standard DHS, 2017-18 | 2329 |
| AGO | AO | Angola Standard DHS, 2015-16 | 4900 |
| ARM | AM | Armenia Standard DHS, 2010 | 1352 |
| | | Armenia Standard DHS, 2015-16 | 1625 |
| BGD | BD | Bangladesh Standard DHS, 2017-18 | 2142 |
| BEN | BJ | Benin Standard DHS, 2011-12 | 5631 |
| | | Benin Standard DHS, 2017-18 | 5916 |
| BFA | BF | Burkina Faso Standard DHS, 2010 | 8946 |
| BDI | BU | Burundi MIS, 2010 | 4425 |
| | | Burundi Standard DHS, 2016-17 | 9756 |
| KHM | KH | Cambodia Standard DHS, 2005 | 1233 |
| | | Cambodia Standard DHS, 2010 | 5116 |
| | | Cambodia Standard DHS, 2014 | 5738 |
| CMR | CM | Cameroon Standard DHS, 2004 | 431 |
| | | Cameroon Standard DHS, 2011 | 6616 |
| | | Cameroon Standard DHS, 2018 | 6049 |
| TCD | TD | Chad Standard DHS, 2014-15 | 1911 |
| COL | CO | Colombia Standard DHS, 2010 | 11221 |
| COD | CD | Congo Democratic Republic Standard DHS, 2007 | 4241 |
| | | Congo Democratic Republic Standard DHS, 2013-14 | 11359 |
| CIV | CI | Cote d'Ivoire Standard DHS, 2011-12 | 3419 |
| DOM | DR | Dominican Republic Special DHS, 2013 | 620 |
| | | Dominican Republic Standard DHS, 2007 | 4462 |
| | | Dominican Republic Standard DHS, 2013 | 2201 |
| EGY | EG | Egypt Standard DHS, 2005 | 1125 |
| | | Egypt Standard DHS, 2008 | 3360 |
| | | Egypt Standard DHS, 2014 | 8273 |
| SWZ | SZ | Eswatini Standard DHS, 2006-07 | 1398 |
| ETH | ET | Ethiopia Standard DHS, 2016 | 1885 |
| GAB | GA | Gabon Standard DHS, 2012 | 3749 |
| GHA | GH | Ghana Standard DHS, 2008 | 959 |
| | | Ghana Standard DHS, 2014 | 2871 |
| GTM | GU | Guatemala Standard DHS, 2014-15 | 11239 |
| GIN | GN | Guinea Standard DHS, 2005 | 594 |
| | | Guinea Standard DHS, 2012 | 2020 |
| | | Guinea Standard DHS, 2018 | 2740 |
| GUY | GY | Guyana Standard DHS, 2009 | 1264 |

| | | | |
|---|---|---|---|
| HTI | HT | Haiti Standard DHS, 2005-2006 | 315 |
| | | Haiti Standard DHS, 2012 | 950 |
| | | Haiti Standard DHS, 2016-17 | 1000 |
| HND | HN | Honduras Standard DHS, 2011-12 | 7235 |
| IND | IA | India Standard DHS, 2015-16 | 182783 |
| | | India Standard DHS, 2019-21 | 89958 |
| JOR | JO | Jordan Standard DHS, 2007 | 7162 |
| | | Jordan Standard DHS, 2012 | 9627 |
| | | Jordan Standard DHS, 2017-18 | 9465 |
| KEN | KE | Kenya Standard DHS, 2008-09 | 2608 |
| | | Kenya Standard DHS, 2014 | 5349 |
| KGZ | KY | Kyrgyz Republic Standard DHS, 2012 | 4085 |
| LSO | LS | Lesotho Standard DHS, 2004 | 478 |
| | | Lesotho Standard DHS, 2009 | 2697 |
| | | Lesotho Standard DHS, 2014 | 2538 |
| LBR | LB | Liberia Standard DHS, 2007 | 331 |
| | | Liberia Standard DHS, 2013 | 1148 |
| | | Liberia Standard DHS, 2019-20 | 636 |
| MDG | MD | Madagascar Standard DHS, 2008-09 | 3961 |
| MWI | MW | Malawi Standard DHS, 2004 | 1045 |
| | | Malawi Standard DHS, 2010 | 11027 |
| | | Malawi Standard DHS, 2015-16 | 12389 |
| MLI | ML | Mali Standard DHS, 2006 | 2097 |
| | | Mali Standard DHS, 2012-13 | 3213 |
| | | Mali Standard DHS, 2018 | 3297 |
| MDA | MB | Moldova Standard DHS, 2005 | 556 |
| MAR | MA | Morocco Standard DHS, 2003-04 | 19 |
| MOZ | MZ | Mozambique Standard DHS, 2011 | 4690 |
| MMR | MM | Myanmar Standard DHS, 2015-16 | 1857 |
| NAM | NM | Namibia Standard DHS, 2006-07 | 2497 |
| | | Namibia Standard DHS, 2013 | 4006 |
| NPL | NP | Nepal Standard DHS, 2006 | 510 |
| | | Nepal Standard DHS, 2011 | 1922 |
| | | Nepal Standard DHS, 2016 | 3067 |
| NER | NI | Niger Standard DHS, 2012 | 3526 |
| NGA | NG | Nigeria Standard DHS, 2008 | 3797 |
| | | Nigeria Standard DHS, 2013 | 4775 |
| | | Nigeria Standard DHS, 2018 | 7121 |
| PAK | PK | Pakistan Standard DHS, 2006-07 | 435 |
| | | Pakistan Standard DHS, 2017-18 | 1330 |
| PER | PE | Peru Continuous DHS, 2009 | 7556 |
| PHL | PH | Philippines Standard DHS, 2008 | 2053 |
| | | Philippines Standard DHS, 2017 | 3695 |

| | | | |
|---|---|---|---|
| RWA | RW | Rwanda Standard DHS, 2005 | 788 |
| | | Rwanda Standard DHS, 2010 | 5425 |
| | | Rwanda Standard DHS, 2014-15 | 6509 |
| | | Rwanda Standard DHS, 2019-20 | 1815 |
| SEN | SN | Senegal Continuous DHS, 2012-13 | 2802 |
| | | Senegal Continuous DHS, 2014-6R | 5247 |
| | | Senegal Continuous DHS, 2014-70 | 2445 |
| | | Senegal Continuous DHS, 2015 | 2632 |
| | | Senegal Continuous DHS, 2016-71 | 2555 |
| | | Senegal Continuous DHS, 2017 | 5333 |
| | | Senegal Continuous DHS, 2018 | 3157 |
| | | Senegal Continuous DHS, 2019 | 3093 |
| | | Senegal Standard DHS, 2005 | 1189 |
| | | Senegal Standard DHS, 2010-11 | 5157 |
| SLE | SL | Sierra Leone Standard DHS, 2008 | 1444 |
| | | Sierra Leone Standard DHS, 2013 | 4565 |
| | | Sierra Leone Standard DHS, 2019 | 4808 |
| TJK | TJ | Tajikistan Standard DHS, 2012 | 3958 |
| | | Tajikistan Standard DHS, 2017 | 5519 |
| TZA | TZ | Tanzania Standard DHS, 2010 | 2750 |
| | | Tanzania Standard DHS, 2015-16 | 4321 |
| TLS | TL | Timor-Leste Standard DHS, 2009-10 | 915 |
| | | Timor-Leste Standard DHS, 2016 | 1547 |
| TGO | TG | Togo Standard DHS, 2013-14 | 3486 |
| UGA | UG | Uganda Standard DHS, 2006 | 1418 |
| | | Uganda Standard DHS, 2011 | 3817 |
| | | Uganda Standard DHS, 2016 | 9196 |
| ZMB | ZM | Zambia Standard DHS, 2007 | 2219 |
| | | Zambia Standard DHS, 2013-14 | 8354 |
| | | Zambia Standard DHS, 2018 | 7209 |
| ZWE | ZW | Zimbabwe Standard DHS, 2005-06 | 1461 |
| | | Zimbabwe Standard DHS, 2010-11 | 3671 |
| | | Zimbabwe Standard DHS, 2015 | 4994 |

**Table S2.** Characteristics of the study population (N = 697,148).

| Characteristic (n, %) | |
|---|---|
| Birthweight [g, mean (sd)] | 3061.5 (678.6) |
| Maternal age [Year, mean (sd)] | 26.00 (6.00) |
| Age of household head [Year, mean (sd)] | 42.91 (14.59) |
| $O_3$ [ppb, mean (sd)] | 52.67 (10.35) |
| $PM_{2.5}$ [$\mu g/m^3$, mean (sd)] | 50.58 (31.13) |
| Temperature [K, mean (sd)] | 298.78 (4.41) |
| Geographical region | |
| East Asia & Pacific | 22154 (3.2) |
| Europe & Central Asia | 20851 (3.0) |
| Latin America & Caribbean | 48063 (6.9) |
| Middle East & North Africa | 39031 (5.6) |
| South Asia | 282147 (40.5) |
| Sub-Saharan Africa | 284902 (40.9) |
| Income group | |
| Upper middle income | 67466 (9.7) |
| Lower middle income | 417061 (59.8) |
| Low income | 212621 (30.5) |
| Sex of infant: Male | 359863 (51.6) |
| Caesarean section | |
| No | 592418 (85.0) |
| Yes | 101763 (14.6) |
| Unknown | 2967 (0.4) |
| Place of delivery | |
| Home | 58851 (8.4) |
| Hospital | 507424 (72.8) |
| Other | 3163 (0.5) |
| Private | 122868 (17.6) |
| Unknown | 4842 (0.7) |
| Antenatal care attendance | |
| No | 503308 (72.2) |
| Yes | 27581 (4.0) |
| Unknown | 166259 (23.8) |
| Nulliparous: Yes | 234956 (33.7) |
| Maternal body mass index | |
| Underweight | 74551 (10.7) |
| Normal | 314543 (45.1) |
| Overweight | 93651 (13.4) |
| Obese | 37513 (5.4) |
| Unknown | 176890 (25.4) |
| Maternal employment | |
| No | 196134 (28.1) |

| | |
|---|---|
| Yes | 263563 (37.8) |
| Unknown | 237451 (34.1) |
| Sex of household head: Male | 575309 (82.5) |
| Sanitation: water source | |
| Bottled | 28368 (4.1) |
| Natural | 72723 (10.4) |
| Other | 7131 (1.0) |
| Piped | 283531 (40.7) |
| Rain | 5130 (0.7) |
| Tank | 10280 (1.5) |
| Tube | 170104 (24.4) |
| Well | 82082 (11.8) |
| Unknown | 37799 (5.4) |
| Sanitation: toilet type | |
| Composting | 10752 (1.5) |
| Flush | 260503 (37.4) |
| No | 152670 (21.9) |
| Other | 1428 (0.2) |
| Pit | 246007 (35.3) |
| Unknown | 25788 (3.7) |
| Type of cooking energy | |
| Agricultural crop | 7684 (1.1) |
| Animal dung | 20430 (2.9) |
| Biogas | 2467 (0.4) |
| Charcoal | 58792 (8.4) |
| Coal, lignite | 13730 (2.0) |
| Electricity | 25296 (3.6) |
| Kerosene | 11574 (1.7) |
| LPG | 147870 (21.2) |
| Natural gas | 24148 (3.5) |
| Other | 1078 (0.2) |
| Straw/shrubs/grass | 23813 (3.4) |
| Wood | 323185 (46.4) |
| Unknown | 37081 (5.3) |

**Table S3.** Description of the ozone exposure (ppb) across the difference regions.

| Ozone exposure | Region | Mean | SD | Median | $P_{25}$ | $P_{75}$ | Min | Max |
|---|---|---|---|---|---|---|---|---|
| Maternal peak season exposure | East Asia & Pacific | 38.54 | 6.94 | 38.67 | 34.48 | 41.68 | 25.51 | 53.33 |
| | Europe & Central Asia | 56.81 | 5.81 | 57.43 | 52.83 | 60.88 | 44.45 | 66.57 |
| | Latin America & Caribbean | 38.70 | 5.95 | 39.43 | 34.41 | 43.45 | 26.32 | 48.10 |
| | Middle East & North Africa | 66.63 | 3.06 | 67.03 | 65.02 | 68.69 | 59.89 | 71.16 |
| | South Asia | 60.29 | 6.40 | 59.89 | 55.96 | 64.49 | 47.56 | 73.15 |
| | Sub-Saharan Africa | 46.37 | 5.88 | 45.95 | 42.54 | 50.41 | 34.92 | 58.23 |
| | All | 52.67 | 10.35 | 52.83 | 44.57 | 60.64 | 33.51 | 71.16 |
| Gestational exposure | East Asia & Pacific | 33.10 | 5.47 | 32.38 | 29.03 | 36.42 | 24.54 | 46.27 |
| | Europe & Central Asia | 49.40 | 6.48 | 49.13 | 44.41 | 54.44 | 37.67 | 61.34 |
| | Latin America & Caribbean | 35.52 | 5.73 | 35.93 | 31.11 | 40.19 | 24.32 | 44.93 |
| | Middle East & North Africa | 57.21 | 3.98 | 56.91 | 54.29 | 60.29 | 50.12 | 64.48 |
| | South Asia | 53.30 | 6.42 | 53.05 | 48.95 | 57.18 | 41.41 | 67.30 |
| | Sub-Saharan Africa | 40.78 | 5.19 | 40.61 | 37.10 | 44.17 | 31.43 | 51.67 |
| | All | 46.42 | 9.26 | 45.88 | 39.43 | 53.52 | 29.62 | 64.40 |
| Yearly peak season exposure | East Asia & Pacific | 31.87 | 6.97 | 32.39 | 27.11 | 35.86 | 18.92 | 45.81 |
| | Europe & Central Asia | 51.42 | 5.35 | 51.60 | 48.82 | 54.85 | 39.36 | 61.35 |
| | Latin America & Caribbean | 32.75 | 7.13 | 33.43 | 27.77 | 37.52 | 18.66 | 46.76 |
| | Middle East & North Africa | 55.20 | 3.89 | 56.04 | 53.55 | 57.48 | 43.40 | 60.65 |
| | South Asia | 60.58 | 7.24 | 61.42 | 55.76 | 65.92 | 44.86 | 73.07 |
| | Sub-Saharan Africa | 38.35 | 6.97 | 38.41 | 33.86 | 42.81 | 24.61 | 52.31 |
| | All | 47.07 | 13.27 | 45.73 | 36.44 | 58.17 | 24.00 | 69.79 |
| Gestation-weighted exposure | East Asia & Pacific | 31.95 | 6.85 | 32.51 | 27.39 | 35.87 | 19.29 | 45.78 |
| | Europe & Central Asia | 51.31 | 5.05 | 51.67 | 48.84 | 54.34 | 39.85 | 60.62 |
| | Latin America & Caribbean | 32.88 | 7.09 | 33.63 | 27.79 | 37.54 | 18.77 | 46.73 |
| | Middle East & North Africa | 55.18 | 3.70 | 55.95 | 53.83 | 57.38 | 43.27 | 60.29 |
| | South Asia | 60.28 | 7.17 | 61.11 | 55.51 | 65.63 | 44.69 | 72.55 |
| | Sub-Saharan Africa | 38.24 | 6.73 | 38.43 | 33.91 | 42.48 | 24.62 | 51.60 |
| | All | 46.92 | 13.10 | 45.25 | 36.51 | 57.86 | 24.05 | 69.42 |

SD, standard deviation; $P_{25}$, lower quantile; $P_{75}$, upper quantile;

**Table S4.** Summaries of ozone ($O_3$) exposure within stratum or between strata.

| Temporal and spatial resolutions | Exposure assessment method | Between strata | | Within stratum | | Fraction of within-stratum variance (%) |
|---|---|---|---|---|---|---|
| | | Mean (ppb) | Variance ($ppb^2$) | Mean (ppb) | Variance ($ppb^2$) | |
| Monthly, 0.5° × 0.5° | Maternal peak-season exposure | 47.11 | 75.99 | 0.00 | 8.53 | 10.1% |
| | Gestational exposure | 41.64 | 54.35 | 0.00 | 12.07 | 18.2% |
| Annual, 0.1° × 0.1° | Maternal peak-season exposure | 38.77 | 92.03 | 0.00 | 14.34 | 13.5% |
| | Gestational exposure | 38.72 | 90.53 | 0.00 | 12.12 | 11.8% |

(a)

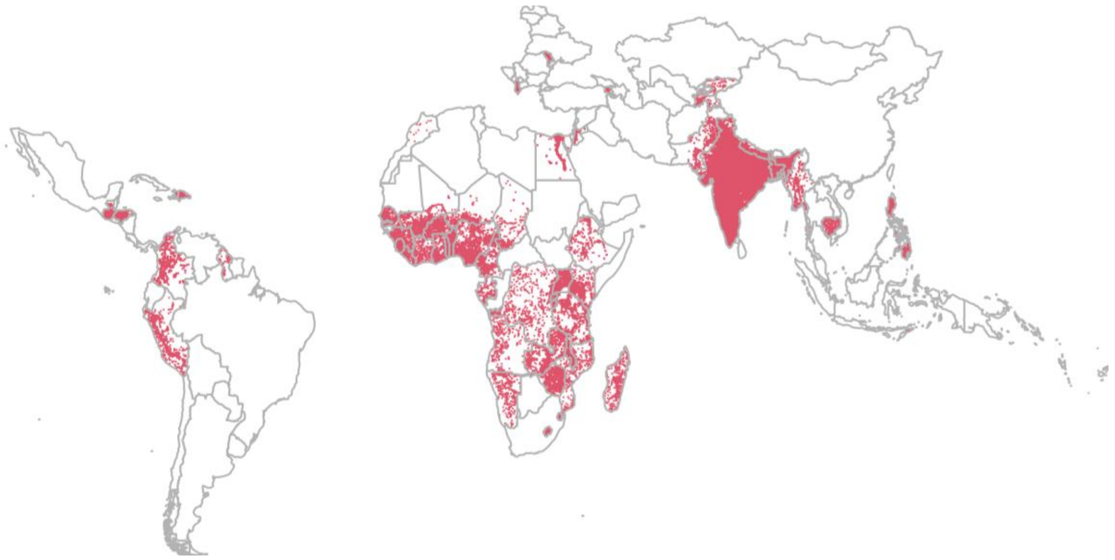

(b)

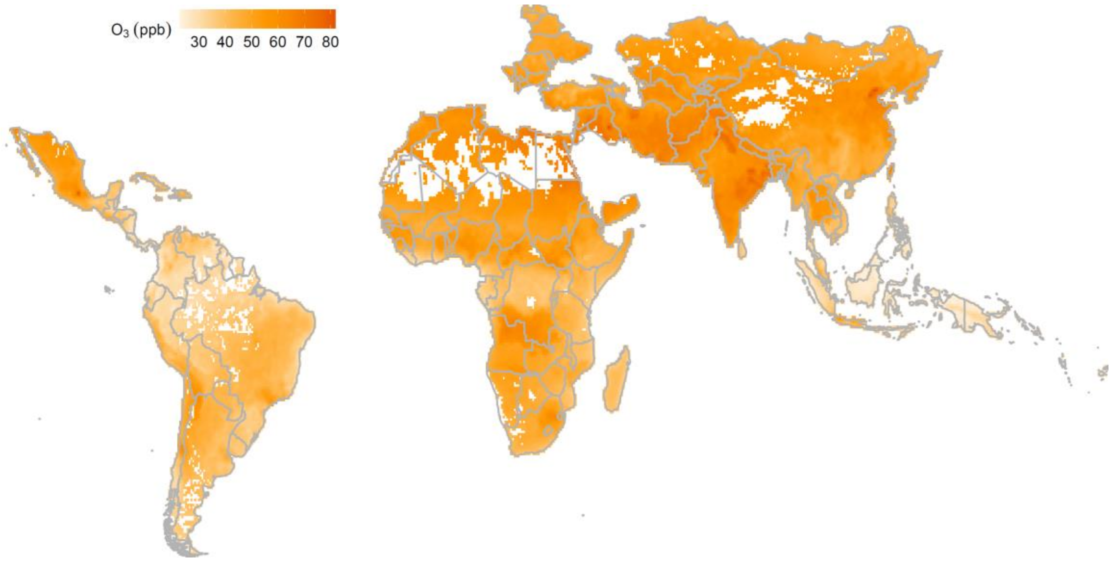


**Fig. S1.** Geographic locations of study population (red points) from 54 low- and middle-income countries (LMICs, a), and study domain for ozone ($O_3$)-related risk assessment in 123 LMICs and average level of peak season ozone during 2003-2019 (b).

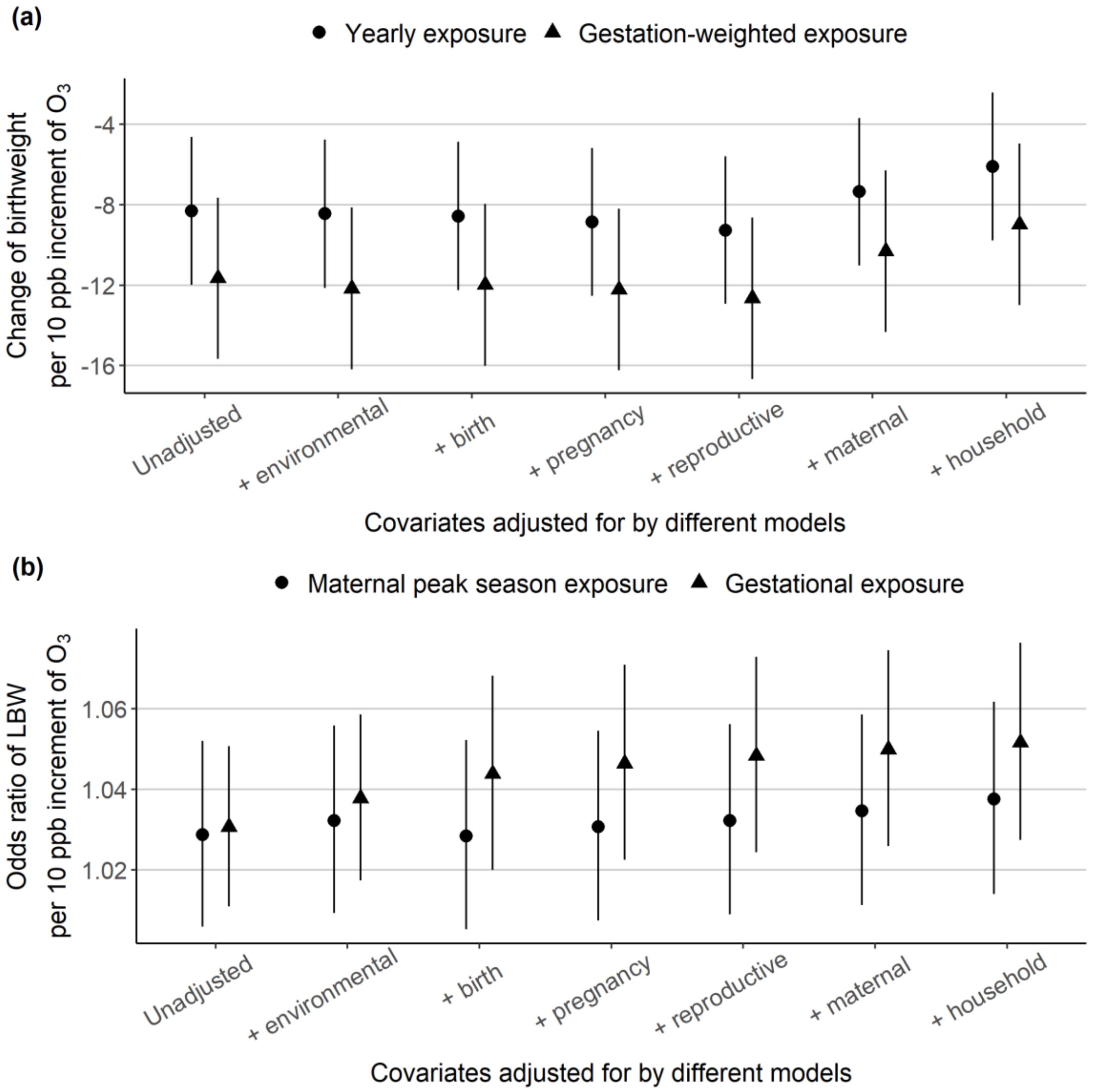


**Fig. S2.** Linear association between ozone ($O_3$) exposure and birthweight estimated by different models. A 0.1° × 0.1° grid $O_3$ concentration was utilized to estimate the exposure level. The adjusted covariates are (1) environmental variables ($PM_{2.5}$ and temperature); (2) birth characteristics (sex and month of birth × latitude zones); (3) pregnancy variables (caesarean section, place of delivery, antenatal care attendance, and nulliparous or not); (4) variables related to reproductive history (maternal age and inter-pregnancy interval); (5) maternal variables (maternal body mass index, and maternal employment status); and (6) household features (sex and age of household head, source of drinking water, type of toilet, and type of cooking energy).

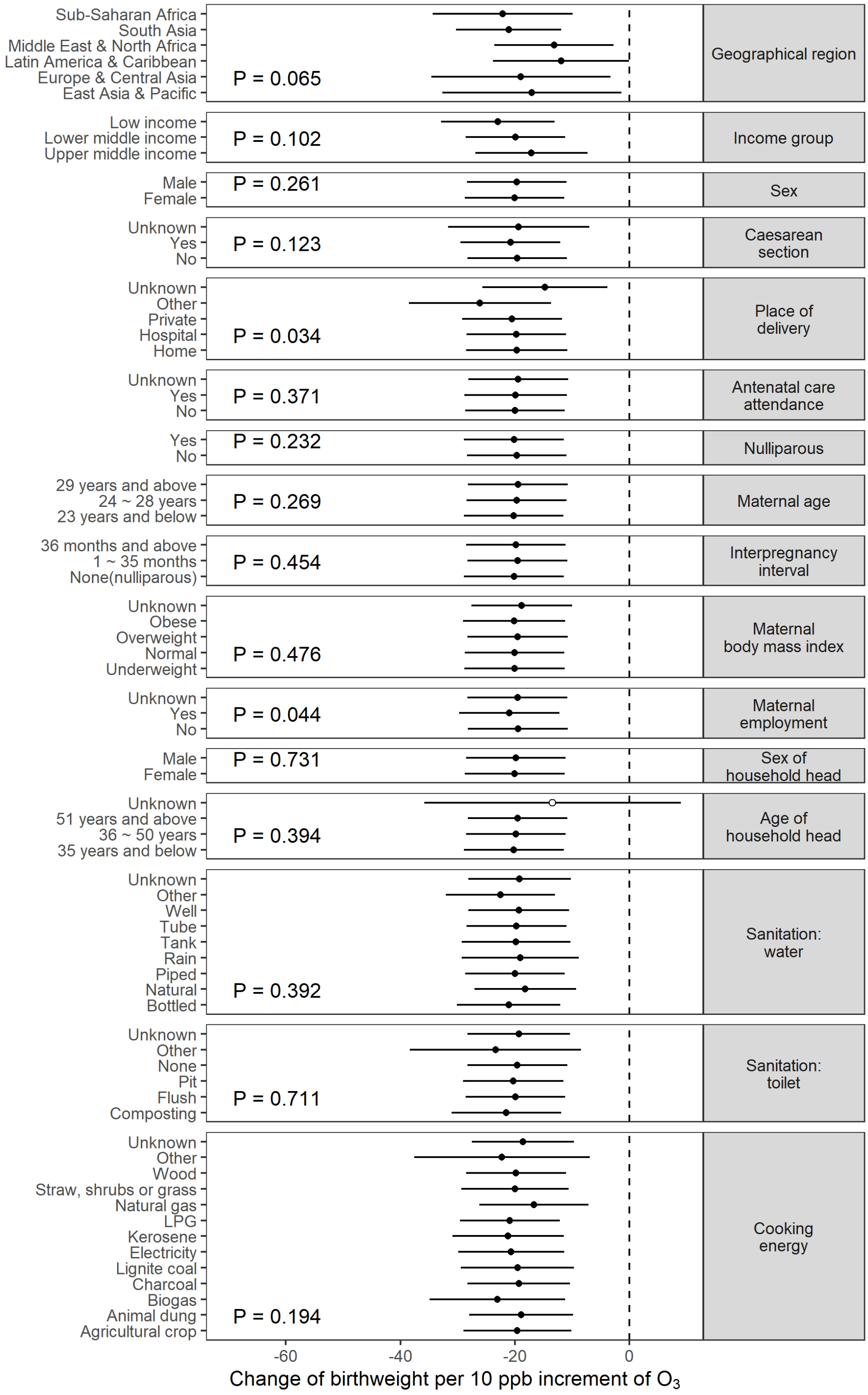


**Fig. S3.** The linear association between maternal peak-season ozone ($O_3$) exposure and birthweight estimated by different subpopulations.

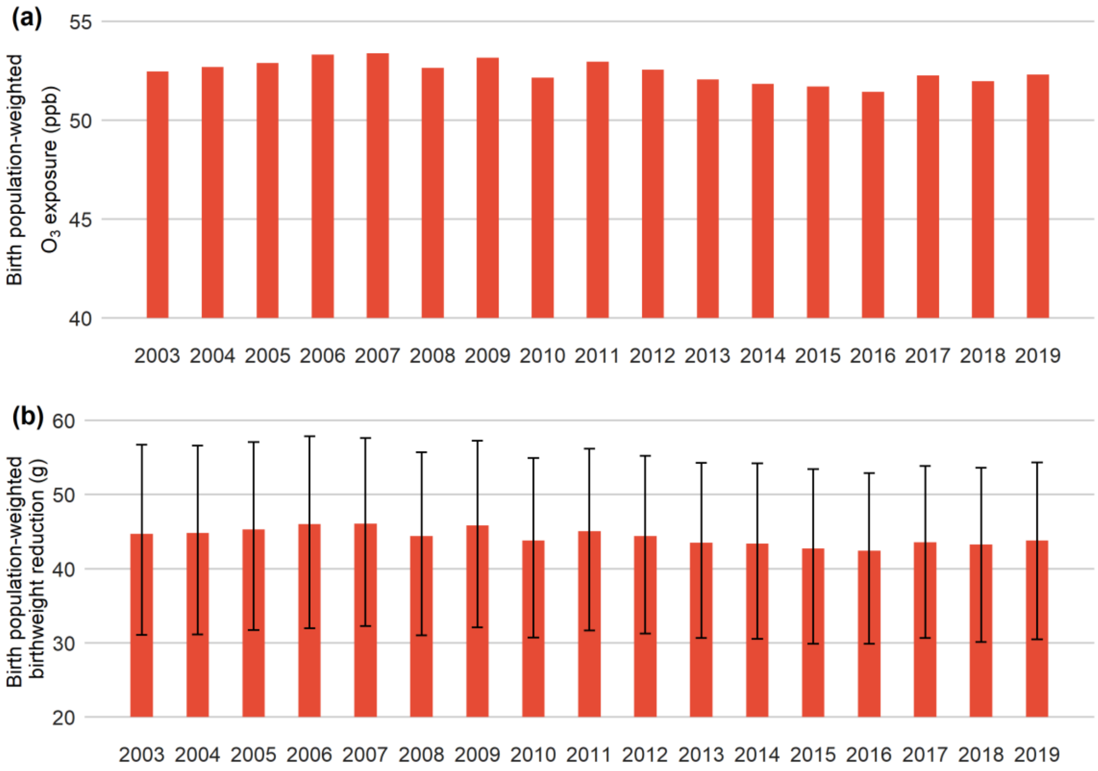


**Fig. S4**. The temporal trends of global birth population-weighted ozone ($O_3$) exposure (a) and global birth population-weighted birthweight reduction attributable to $O_3$ (b).

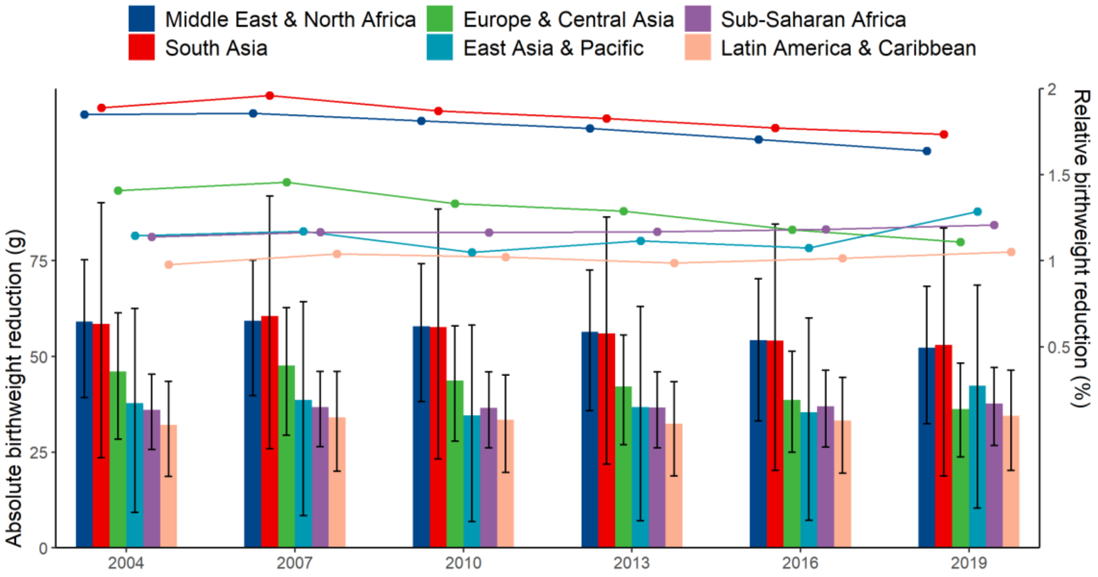


**Fig. S5.** The temporal trends of absolute and relative birthweight reduction attributable to ozone ($O_3$) exposure by different regions.
